# Correction: Hoffmann et al. Detecting Walking Challenges in Gait Patterns Using a Capacitive Sensor Floor and Recurrent Neural Networks. *Sensors* 2021, *21*, 1086

**DOI:** 10.3390/s22134896

**Published:** 2022-06-29

**Authors:** Raoul Hoffmann, Hanna Brodowski, Axel Steinhage, Marcin Grzegorzek

**Affiliations:** 1 SensProtect GmbH, 85635 Höhenkirchen-Siegertsbrunn, Germany; axel.steinhage@future-shape.com; 2 Institute of Medical Informatics, University of Lübeck, 23538 Lübeck, Germany; grzegorzek@imi.uni-luebeck.de; 3 Institute of Health Sciences, Department of Physiotherapy, Pain and Exercise Research Lübeck (P.E.R.L.), University of Lübeck, 23538 Lübeck, Germany; hanna.brodowski@uni-luebeck.de; 4 Geriatrics Research Group, Charité-Universitätsmedizin Berlin, 13347 Berlin, Germany

There was an affiliation missing in the original article [1]. For the author Hanna Brodowski, we added the following affiliation: Geriatrics Research Group, Charité-Universitätsmedizin Berlin, 13347 Berlin, Germany.

The authors apologize for any inconvenience caused, and state that the scientific conclusions are unaffected. The original article has been updated.
